# A Retrospective Analysis of Thromboembolic Phenomena in Mechanically Ventilated Patients with COVID-19

**DOI:** 10.1155/2021/8737580

**Published:** 2021-01-08

**Authors:** Fahad Faqihi, Abdulrahman Alharthy, Abdullah Balhamar, Nasir Nasim, Khaled Alanezi, Feisal Alaklobi, Ziad A. Memish, Mike Blaivas, Saleh A. Alqahtani, Dimitrios Karakitsos

**Affiliations:** ^1^Critical Care Department, King Saud Medical City, Riyadh, Saudi Arabia; ^2^King Saud Medical City, Riyadh, Saudi Arabia; ^3^Research and Innovation Center, King Saud Medical City, Riyadh, Saudi Arabia; ^4^Department of Internal Medicine, University of South Carolina, School of Medicine, Columbia, SC, USA; ^5^Transplant Hepatology Division of Gastroenterology and Hepatology, Johns Hopkins University, School of Medicine, Baltimore, MD, USA

## Abstract

**Background:**

Recent studies have shown an increased prevalence of thromboembolic disease in critically ill patients with the novel SARS-CoV-2 disease (COVID-19). However, the use of enhanced anticoagulation therapy in these patients remains controversial.

**Objectives:**

To determine the incidence of thromboembolic phenomena (TEP) and hemorrhagic events (HEs) in intensive care unit (ICU) COVID-19 patients.

**Methods:**

One hundred and sixty ICU patients with COVID-19 were enrolled. Clinical examination results, laboratory data, and imaging studies (computed tomography/Doppler ultrasound scans) for these patients were retrospectively collected and analyzed. Outcome measures including days on mechanical ventilation, ICU length of stay, and day-28 mortality were recorded.

**Results:**

Sixty patients (37.5%) developed TEP including thirty patients with deep vein thrombosis, 55 patients with pulmonary embolism, and 7 patients with arterial thromboembolism. Cardiac arrhythmias, lymphocytopenia, and increased D-dimers were more frequently observed in the TEP group compared to the non-TEP group of patients (all *p* < 0.05). The sensitivity, specificity, and positive and negative predictive values of a cutoff D-dimer level of 3.0 *μ*g/mL for predicting PE were 74.5%, 95.1%, 86.8%, and 91.9%, respectively. Thirteen patients experienced HEs, which were more frequently observed in the TEP group (*p* < 0.05). Twenty-eight-day mortality was higher in the TEP group (60%) compared to the non-TEP group (30%) of patients (*p*=0.02).

**Conclusions:**

The rates of TEP and HEs in mechanically ventilated critically ill COVID-19 patients were 37. 5% and 8.1%. Twenty-eight-day mortality was higher in the TEP group (60%) compared to the non-TEP group (30%) of patients.

## 1. Introduction

The novel coronavirus SARS-CoV-2 disease (COVID-19) emerged in Wuhan city, the capital of Hubei Province in China, and has progressively spread throughout the world. A minority of patients can develop life-threatening disease, which is characterized by acute respiratory distress syndrome (ARDS), sepsis, multisystem organ failure, neurological and other extrapulmonary manifestations, and thromboembolic disease [1].

An increased prevalence of pulmonary embolism (PE) in critically ill COVID-19 patients was previously reported [2,3]. Also, the administration of enhanced anticoagulation in patients with severe COVID-19 and Padua prediction score ≥4 or D-dimers >3.0 *μ*g/mL was suggested [4]. Recently, we documented a PE rate of approximately 25% in a point-of-care ultrasound study in critically ill COVID-19 patients [5]. Herein, we present a brief retrospective analysis of thromboembolic phenomena (TEP) in mechanically ventilated critically ill COVID-19 patients.

## 2. Methods

We retrospectively analyzed COVID-19 patients that were admitted to our 300-bed intensive care unit (ICU) between April 1 and May 30, 2020. All patients were diagnosed according to the World Health Organization guidelines [6]. COVID-19 was confirmed by real-time polymerase chain reaction (RT-PCR) assays, using QuantiNova Probe RT-PCR kit (Qiagen, GmbH, Germany) in a light-cycler 480 real-time PCR system (Roche, Basel, Switzerland), which were performed on nasopharyngeal swabs [7]. The coagulation profiles of patients, which included prothrombin time, activated partial thromboplastin time, thrombin time, international normalized ratio, fibrinogen, and D-dimers, were performed using an automatic coagulation analyzer (Sysmex CS-2500 System, Siemens, Germany) and were retrieved from their electronic medical record files. Upon ICU admission, the Sequential Organ Function Assessment (SOFA) and the Acute Physiology and Chronic Health Evaluation II (APACHE II) scores were included in the analysis. Researchers recorded all TEP during the patients' ICU length of stay such as PE, deep vein thrombosis, and arterial thromboembolism based on clinical examinations, chest computed tomography (CT), CT angiography, and lower limb venous Doppler ultrasound. Hemorrhagic events (HEs) defined as pause of anticoagulation therapy with blood product transfusion and surgical intervention/death were registered. Outcome measures such as days on mechanical ventilation, ICU length of stay, and 28-day mortality were included in the analysis. All data were retrieved from the patients' records and were retrospectively analyzed. The study was conducted according to the principles outlined in the Declaration of Helsinki and was approved by our Institutional Review Board.

## 3. Statistical Analysis

Continuous variables are expressed as medians with interquartile ranges (IQRs) and categorical variables as absolute numbers/ratios. We utilized the Wilcoxon signed rank test for nonparametric data for group comparisons. All tests were two-tailed and considered significant when the *p* value was <0.05. Analysis was performed using SPSS, version 23.0.

## 4. Results

Of the total two hundred and twenty ICU patients that were admitted during the study period, only one hundred and sixty were included in the final analysis. Sixty patients were excluded as they were transferred to other COVID-19 centers according to our Ministry of Health surge plan. The main characteristics of these one hundred and sixty patients are illustrated in Table 1. The vast majority of our patients were males (125 out of 160) and relatively young as their median age was 49 (IQR: 39–58). Upon ICU admission, all patients had increased SOFA and APACHE II scores. All patients had ARDS and were mechanically ventilated (Table 1). We administered ARDS-net/prone positioning ventilation and empiric treatment with ribavirin, interferon beta-1b, antibiotics, and ICU supportive care to all patients [8]. All mechanically ventilated COVID-19 patients received intermittent pneumatic compression and baseline weight- and renal function-adjusted doses of low-molecular-weight heparin thromboprophylaxis unless contraindicated (enoxaparin 20 mg once daily if <50 kg, enoxaparin 40 mg once daily if 50–100 kg, 40 mg twice daily if 101–150 kg, and 60 mg twice daily if >150 kg) as per hospital protocol [8]. Upon ICU admission (baseline), no hemorrhagic events were recorded; however, 8 out of 160 patients had a history of thromboembolic disease (Table 1).

During hospitalization, sixty patients (37.5%) developed TEP including thirty patients with deep vein thrombosis, fifty-five patients with PE, and seven patients with arterial thromboembolism. These patients received treatment-dose systemic anticoagulation based on the individual case scenario and as per hospital protocol [8]. None of the patients had evidence of developing diffuse intravascular coagulopathy (data not included in Table 1). Specifically, platelet count, prothrombin time, activated partial thromboplastin time, thrombin time, and international normalized ratio did not significantly differ during the study period. Also, no other systemic disorders (i.e., autoimmune disorders) and/or thrombophilia were detected. Parameters that were more frequently observed in the TEP group versus the non-TEP group were as follows: history of cardiac arrhythmias, lymphocytopenia, and increased levels of D-dimer (all *p* < 0.05, Table 1). PE was confirmed by contrast chest CT, which revealed five patients with massive PE, forty patients with subsegmental PE, and ten patients with pulmonary vascular microthrombosis. Using a previously suggested cutoff D-dimer value of 3.0 *μ*g/mL for predicting PE, our analysis revealed that sensitivity, specificity, and positive and negative predictive values were 74.5%, 95.1%, 86.8%, and 91.9%, respectively. In the TEP group, after the administration of anticoagulation therapy, D-dimers gradually decreased over a period of two weeks (from 5.1 (IQR: 2.2–9.1) to 1.6 (IQR: 0.7–1.9); *p*=0.001, Wilcoxon signed rank test)). Mortality on day 28 was higher in the TEP group (60%) compared to the non-TEP group (30%) of patients (*p*=0.02, Table 1).

Thirteen patients developed hemorrhagic events, which were more frequently observed in patients with TEP receiving therapeutic anticoagulation (*p* < 0.05, Table 1). The characteristics of these thirteen patients with hemorrhagic events are illustrated in Table 2. Anticoagulation was interrupted in all cases. Major hemorrhagic events requiring transfusion of blood products were observed in the TEP group of patients. Seven out of thirteen cases required transfusion of blood products, while in four cases, massive transfusion protocol (transfusion of 4 units of red blood cells in less than 4 hours, plus hemodynamic instability and ongoing bleeding) was activated (Table 2). However, anticoagulation was resumed in seven patients approximately 5 (IQR: 2–7) days after the HE. Serial ultrasound surveillance for detection of deep vein thrombosis was applied on all cases. Four patients of the TEP group underwent inferior vena cava filter placement. Finally, two out of the thirteen cases (TEP group) were fatal due to intracranial hemorrhage.

## 5. Discussion

This retrospective study has several limitations, which prevent its generalizability. However, we confirmed the increased incidence of TEP in mechanically ventilated critically ill COVID-19 patients as other studies previously reported [2–4]. Of note, we found a higher rate of cardiac arrhythmias, lymphocytopenia, and increased D-dimers in patients with TEP [1,9,10]. Hypercoagulability with associated vascular dysfunction and ensuing cytokine release syndrome might be the underlying mechanism promoting thromboinflammation in complex COVID-19 critical illness [9–12]. Also, the fact that SARS-CoV-2 can bind to the ACE-2 receptors that are expressed on type II pneumocytes and vascular endothelial cells within the lung could reflect direct injury to the pulmonary vasculature [13]. This vascular pathology along with the parenchymal lung injury (“dual-hit” underlying mechanism) could be at least partially responsible for the refractory ARDS observed in critically ill patients with COVID-19 [14].

In this study (April to May 2020), the increased observed prevalence of PE as compared to our previously reported data (lower PE rate) for the month of June 2020 could be explained by the fact that the retrospective data were derived from the early stages of the COVID-19 outbreak in our country [5]. Hence, the observed discrepancies could reflect the change in our practices such as the integration of a more robust anticoagulation protocol for critically ill COVID-19 patients and better surveillance for the development of TEP [5,8].

We found that an elevated D-dimer level >3.0 *μ*g/mL could predict the development of PE in mechanically ventilated COVID-19 patients with a sensitivity of 74.5% and a specificity of 95.1%. Moreover, D-dimer levels gradually decreased after the administration of anticoagulation therapy. Hence, upgrading the systemic anticoagulation in mechanically ventilated patients with life-threatening COVID-19 may be a step in the right direction [4]. However, patients receiving enhanced therapeutic anticoagulation could develop major hemorrhagic events, and therefore, close ICU monitoring is warranted as the risk is not negligible. In this study, a more meaningful subgroup analysis of patients who developed hemorrhagic events was not feasible due to the small number of cases. We speculate that given the absence of such events upon ICU admission (baseline), their development was linked to the administration of therapeutic anticoagulation although the presence of COVID-19-associated coagulopathy cannot be excluded.

In this series, COVID-19-associated coagulopathy was not clearly recorded; however, the latter may exhibit overlapping features of hemophagocytic syndrome, antiphospholipid antibodies, and thrombotic microangiopathy [15–17]. The presence of severe coagulopathy may complicate the administration of therapeutic anticoagulation in critically ill COVID-19 patients although thromboembolic disease per se appears to be more common in COVID-19 compared to sepsis-induced coagulopathy. Despite the aforementioned considerations, the increased mortality rate in mechanically ventilated COVID-19 patients with TEP was consistent with recently published data [18].

## 6. Conclusion

We found that the rate of thromboembolic phenomena and hemorrhagic events in critically ill patients with COVID-19 was 37. 5% and 8.1%, respectively. D-Dimer level can be used in identifying high risk patients for developing TEP. Finally, the optimal anticoagulation regime [19], the interactions between anticoagulants and antivirals, and the follow-up prophylaxis with new oral anticoagulants [20] based on risk stratification algorithms could be explored in future prospective studies.

## Figures and Tables

**Table 1:** Characteristics of critically ill patients with COVID-19 who developed (*n* = 60, 37.5%) or not (*n* = 100, 62.5%) thromboembolic phenomena.

Parameters	All patients, *n* = 160	Patients without TEP, *n* = 100 (62.5%)	Patients with TEP, *n* = 60 (37.5%)	*p* value
Age (years)	49 (39–58)	49 (38–59)	50 (39–58)	0.9
Sex (males/females)	125/35	80/20	45/15	0.6
Body mass index (kg/m²)	27 (21–32)	27 (20–32)	28 (20–32)	0.45
Baseline SOFA score	6 (4–10)	6 (3–9)	7 (4–11)	0.25
Baseline APACHE II score	19 (16–23)	18 (16–22)	19 (17–23)	0.19
*Comorbidities*				
Hypertension (yes/no)	100/60	60/40	40/20	0.6
Diabetes mellitus (yes/no)	70/90	40/60	30/30	0.5
Cardiovascular disease (yes/no)	30/130	20/80	10/50	0.45
Cardiac arrhythmias (yes/no)	16/144	4/96	12/48	0.04^*∗*^
Thromboembolic disease (yes/no)	8/152	5/95	3/57	0.5
Chronic respiratory disorder (yes/no)	18/142	10/90	8/52	0.45
End-stage kidney disease (yes/no)	14/146	8/92	6/54	0.5
*Baseline hematological profile*				
Platelets (G/L)	229 (207–291)	228 (213–256)	231 (207–268)	0.56
Fibrinogen (g/L)	7.5 (5.3–9)	7.7 (5.5–8.9)	7.6 (5.2–8.7)	0.35
D-Dimers (*μ*g/ml)	2.7 (1.2–8.9)	2.8 (1.2–5.5)	5.1 (2.2–9.1)	0.02^*∗*^
White blood cells (10^9^/L)	20 (16–24)	19 (14–23)	19 (16–24)	0.75
Lymphocytes (10^9^/L)	0.99 (0.71–1.2)	0.98 (0.77 to 1.2)	0.75 (0.45 to 1.1)	0.035^*∗*^
*Anticoagulation therapy*				
Prophylactic anticoagulation	160/160	100/100	60/60	0.9
Therapeutic anticoagulation	60/160	0/100	60/60	0.001^^*∗*^^
*Thromboembolic phenomena*				
Deep vein thrombosis (yes/no)	30/130	0/100	30/30	0.001^*∗*^
Pulmonary thromboembolism (yes/no)	55/105	0/100	55/5	0.001^*∗*^
Arterial thromboembolism (yes/no)	7/153	0/100	7/53	0.04^*∗*^
Hemorrhagic events (yes/no)	13/147	3/97	10/50	0.045^*∗*^
*Outcome measures*				
Days on mechanical ventilation	18 (14–26)	18 (12–25)	20 (14–29)	0.35
ICU length of stay	21 (16–28)	20 (16–26)	21 (17–30)	0.35
Extracorporeal membrane oxygenation	7/153	4/96	3/57	0.6
Continuous renal replacement therapy	19/141	10/90	9/51	0.35
Mortality on day 28 post-ICU admission (*n*, %)	54 (33.75%)	30 (30%)	24 (60%)	0.02^*∗*^

TEP = thromboembolic phenomena; ICU = intensive care unit; APACHE II score = Acute Physiology and Chronic Health Evaluation II score; SOFA score = Sequential Organ Function Assessment score. Values are medians with interquartile ranges. ^*∗*^*p* values <0.05 were statistically significant (Wilcoxon signed rank test).

**Table 2:** Characteristics of thirteen critically ill COVID-19 patients who developed hemorrhagic events.

Parameters	Patients without TEP, *n* = 3	Patients with TEP, *n* = 10
Age (years)	55 (53–57)	56 (54–60)
Sex (males/females)	1/2	6/4
Body mass index (kg/m²)	29 (27–31)	30 (28–32)
Baseline SOFA score	7 (6–9)	8 (7–10)
Baseline APACHE II score	20 (19–21)	21 (19–23)
*Comorbidities*		
Hypertension (yes/no)	3/0	8/2
Diabetes mellitus (yes/no)	1/2	5/5
Cardiovascular disease (yes/no)	1/2	5/5
Cardiac arrhythmias (yes/no)	1/2	5/5
Thromboembolic disease (yes/no)	—	2/8
Chronic respiratory disorder (yes/no)	—	4/6
End-stage kidney disease (yes/no)	—	2/8
*Hemorrhagic events and management*		
Minor hemorrhagic events (no transfusion, *n*)	2	3
Major hemorrhagic events (transfusion required, *n*)	—	7
Anticoagulation interrupted (*n*)	3	7
Gastrointestinal bleeding requiring endoscopy (*n*)	—	2
Intracranial hemorrhage (*n*)	—	2
Massive transfusion protocol activated (*n*)^*∗*^	—	4
Inferior vena cava filter placed (*n*)	—	4
Serial ultrasound surveillance for DVT (*n*)	3	10
Fatalities (*n*)	—	2

TEP = thromboembolic phenomena; ICU = intensive care unit; APACHE II score = Acute Physiology and Chronic Health Evaluation II score; SOFA score = Sequential Organ Function Assessment score; DVT: deep vein thrombosis. ^*∗*^Massive transfusion was defined as replacement of >1 blood volume in 24 hours or >50% of blood volume in 4 hours (adult blood volume is approximately 70 mL/kg).

## Data Availability

All pertinent data sets included in this study are available from the corresponding author on reasonable request.

## Abbreviations

SARS-CoV-2 disease: COVID-19
TEP: Thromboembolic phenomena
HE: Hemorrhagic events
ICU: Intensive care unit
CT: Computed tomography
PE: Pulmonary embolism
SOFA score: Sequential Organ Function Assessment score
APACHE II score: Acute Physiology and Chronic Health Evaluation II score
ARDS: Acute respiratory distress syndrome.

## Additional Points

*Highlights.* We observed that the rates of TEP and HEs in mechanically ventilated critically ill COVID-19 patients were 37.5% and 8.1%. Twenty-eight-day mortality was higher in the TEP group (60%) compared to the non-TEP group (30%) of patients. The sensitivity, specificity, and positive and negative predictive values of a cutoff D-dimer level of 3.0 *μ*g/mL for predicting pulmonary embolism in critically ill COVID-19 patients were 74.5%, 95.1%, 86.8%, and 91.9%, respectively. Enhanced anticoagulation in critically ill COVID-19 patients with Padua prediction score ≥4 or D-dimers >3.0 *μ*g/mL may be warranted; however, the administration of enhanced therapeutic anticoagulation requires close monitoring as the risk for hemorrhage is not negligible.
